# 3-(6-Phenylimidazo [2,1-*b*][1,3,4]thiadiazol-2-yl)-1*H*-Indole Derivatives as New Anticancer Agents in the Treatment of Pancreatic Ductal Adenocarcinoma

**DOI:** 10.3390/molecules25020329

**Published:** 2020-01-14

**Authors:** Stella Cascioferro, Giovanna Li Petri, Barbara Parrino, Btissame El Hassouni, Daniela Carbone, Vincenzo Arizza, Ugo Perricone, Alessandro Padova, Niccola Funel, Godefridus J. Peters, Girolamo Cirrincione, Elisa Giovannetti, Patrizia Diana

**Affiliations:** 1 Dipartimento di Scienze e Tecnologie Biologiche Chimiche e Farmaceutiche (STEBICEF), Università degli Studi di Palermo, Via Archirafi 32, 90123 Palermo, Italy; stellamaria.cascioferro@unipa.it (S.C.); giovanna.lipetri@unipa.it (G.L.P.); barbara.parrino@unipa.it (B.P.); daniela.carbone@unipa.it (D.C.); vincenzo.arizza@unipa.it (V.A.); girolamo.cirrincione@unipa.it (G.C.); 2 Department of Medical Oncology, VU University Medical Center, Cancer Center Amsterdam, DeBoelelaan 1117, 1081HV Amsterdam, The Netherlands; b.elhassouni@amsterdamumc.nl (B.E.H.); gj.peters@amsterdamumc.nl (G.J.P.); 3 Fondazione RI.MED, Via Bandiera 11, 90133 Palermo, Italy; uperricone@fondazionerimed.com (U.P.); apadova@fondazionerimed.com (A.P.); 4 Cancer Pharmacology Lab, Fondazione Pisana per la Scienza, via Ferruccio Giovannini 13, 56017 San Giuliano Terme, Pisa, Italy; niccola.funel@gmail.com

**Keywords:** imidazo[2,1-*b*][1,3,4]thiadiazole derivatives, antiproliferative activity, migration assay, indole compounds, pancreatic cancer, resistance

## Abstract

A new series of imidazo[2,1-*b*][1,3,4]thiadiazole derivatives was efficiently synthesized and screened for their in vitro antiproliferative activity on a panel of pancreatic ductal adenocarcinoma (PDAC) cells, including SUIT-2, Capan-1 and Panc-1. Compounds 9c and 9l, showed relevant in vitro antiproliferative activity on all three pre-clinical models with half maximal inhibitory concentration (IC_50_) ranging from 5.11 to 10.8 µM, while the compounds 9e and 9n were active in at least one cell line. In addition, compound 9c significantly inhibited the migration rate of SUIT-2 and Capan-1 cells in the scratch wound-healing assay. In conclusion, our results will support further studies to increase the library of imidazo [2,1-*b*][1,3,4] thiadiazole derivatives for deeper understanding of the relationship between biological activity of the compounds and their structures in the development of new antitumor compounds against pancreatic diseases.

## 1. Introduction

Pancreatic ductal adenocarcinoma (PDAC) is a fatal disease with an increased incidence also in young adults and a mortality/incidence ratio around 98% [1,2]; therefore new therapeutic strategies to counteract this malignancy are urgently needed.

[1,3,4]Thiadiazole, both uncondensed or annealed to other heterocyclic moieties, particularly with an imidazole ring, has been recognized as a valuable scaffold for the development of pharmacologically active derivatives as it is present in many molecules with biological properties including anti-inflammatory [3], anti-Alzheimer’s disease [4], anti-leishmanial [5], antioxidant [6], antitubercular [7], anticonvulsant [8], antibacterial and antibiofilm [9,10,11]. In particular, this ring system showed interesting antitumor activity with different mechanisms of action [12,13,14].

**Figure 1:**
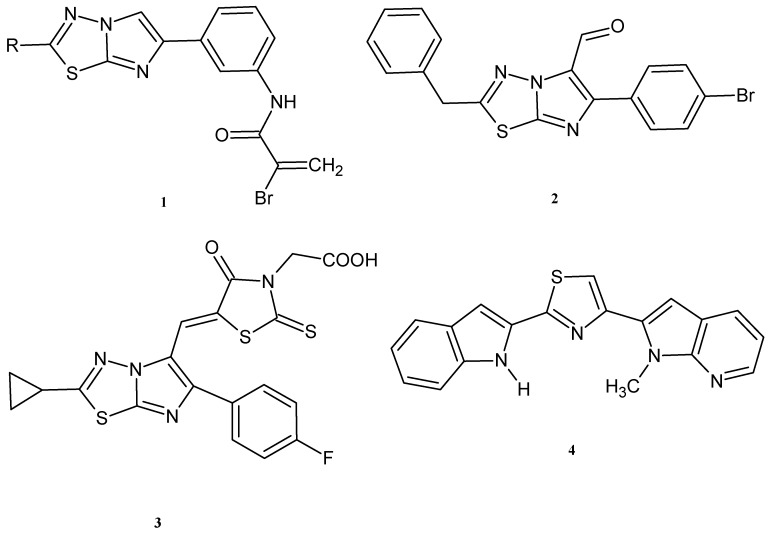
Chemical structures of the imidazo[2,1-*b*][1,3,4]thiadiazole compounds **1**–**3** and of the indole derivative **4**.

Among the imidazo[2,1-*b*][1,3,4]thiadiazole derivatives endowed with antiproliferative properties, the 2,6-disubstituted and the 2,5,6-trisubstituted showed the most interesting activity. Romagnoli et al. reported a series of 2-substituted-6-[*m*-(*α*-bromoacryloylamido)phenyl]imidazo[2,1-*b*][1,3,4]thiadiazoles **1** (Figure 1) with remarkable antiproliferative activity against murine leukaemia (L1210), murine mammary carcinoma (FM3A), human T-lymphocyte leukaemia (CEM) and human cervix carcinoma (HeLa) cells, with IC_50_ values in the submicromolar and nanomolar range [15]. Compound **2** (Figure 1) showed potent cytotoxic activity against the full NCI-60 cell panel eliciting GI_50_ values ranging from 1.4 to 4.2 µM [16]. Another representative example of imidazo[2,1-*b*][1,3,4]thiadiazole derivatives endowed with anticancer activity was reported by Patel and co-workers, who described the outstanding inhibitory activity (IC_50_ = 1.2 nM) of compound **3** (Figure 1) against transforming growth factor-β type I receptor, also known as activin receptor-like kinase 5 [17].

Our previous studies on nitrogen heterocyclic systems with anticancer properties [18,19,20,21,22,23,24,25,26,27,28] and the interesting antiproliferative activity described for the imidazo[2,1-*b*][1,3,4]thiadiazole scaffold encouraged us to continue this approach. Moreover, indole compounds were widely described for their anticancer properties. Among them, the bisindole derivative **4** was reported as a potent antitumor compound, which was able to inhibit CDK1 with an IC_50_ value of 0.86 µM. Therefore, on the basis of the significant antiproliferative activities described for the imidazo[2,1-*b*][1,3,4]thiadiazole and the indole scaffolds, we decided to synthesize and evaluate the cytotoxic activity of a new series of 3-(6-phenylimidazo[2,1-*b*][1,3,4]thiadiazol-2-yl)-1*H*-indole derivatives.

## 2. Results and Discussion

### 2.1. Chemistry

**Scheme 1:**
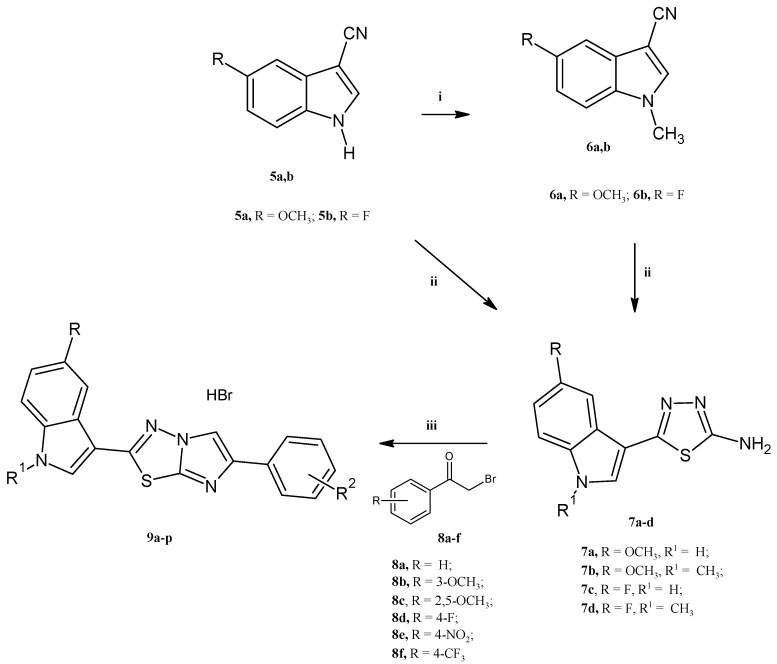
Synthesis of the 3-(6-phenylimidazo[2,1-*b*][1,3,4]thiadiazol-2-yl)-1*H*-indole derivatives **9a**–**p**. Reagents and conditions: (i) DMF, (CH_3_O)_2_CO, K_2_CO_3_, 130 °C, 3.5 h (98%); (ii) trifluoroacetic acid, thiosemicarbazide, 60 °C, 3.5 h (98%); (iii) anhydrous ethanol, reflux, 24 h (60–81%).

A series of new 16 imidazo[2,1-*b*][1,3,4]thiadiazole derivatives **9a**–**p** was synthesized as described in Scheme 1.

The indole-3-carbonitrile **5** was prepared by the reaction of the commercially available 1*H*-indoles with chlorosulfonyl isocyanate (CSI) in anhydrous acetonitrile under stirring at 0 °C (yield 98%) as previously described [10]. The methylation of compounds **5** with dimethyl carbonate in anhydrous DMF at 130 °C afforded the derivatives **6** (yield 98%). The key intermediates 5-(1*H*-indol-3-yl)-1,3,4-thiadiazol-2-amines **7** were prepared in excellent yields (98%) by heating at 60 °C under stirring derivatives **5** or **6** with thiosemicarbazide in trifluoroacetic acid (TFA) for 3.5 h. Finally, by refluxing in anhydrous ethanol the 5-(1*H*-indol-3-yl)-1,3,4-thiadiazol-2-amines **7** with the suitable α-bromoacetyl derivatives **8**, the desired compounds **9** were obtained as hydrobromide salts in good yields (60–81%) (Table 1).

### 2.2. Biological Studies

#### 2.2.1. Antiproliferative Activity of the New Imidazothiadiazole Compounds **9a**–**p** on SUIT-2, Capan-1 and Panc-1 Pancreatic Cancer Cells

The in vitro antiproliferative activity of a new library of imidazo[2,1-*b*][1,3,4]thiadiazole compounds **9a–p** was evaluated by Sulforhodamine-B assay (SRB) on a panel of PDAC cells, including SUIT-2, Capan-1 and Panc-1. These cells have been used in a number of pharmacological studies and are representative of the typical inherent resistance of pancreatic cancer cells to most chemotherapeutic agents [29,30].

All compounds (**9a–p**) were initially screened at three different concentration (0.1, 1 and 16 μM) in all cell lines. The compounds **9c**, **9e**, **9l** and **9n** emerged for their ability to inhibit the growth rate in one or more cell lines. Therefore, these compounds were selected for further screening using eight different increasing concentrations (in the range between 0.125 and 16 µM) in order to evaluate the half maximal inhibitory concentration (IC_50_) values. Table 2 summarizes the IC_50s_ reported as means ± SEM of three independent experiments.

**Figure 2:**
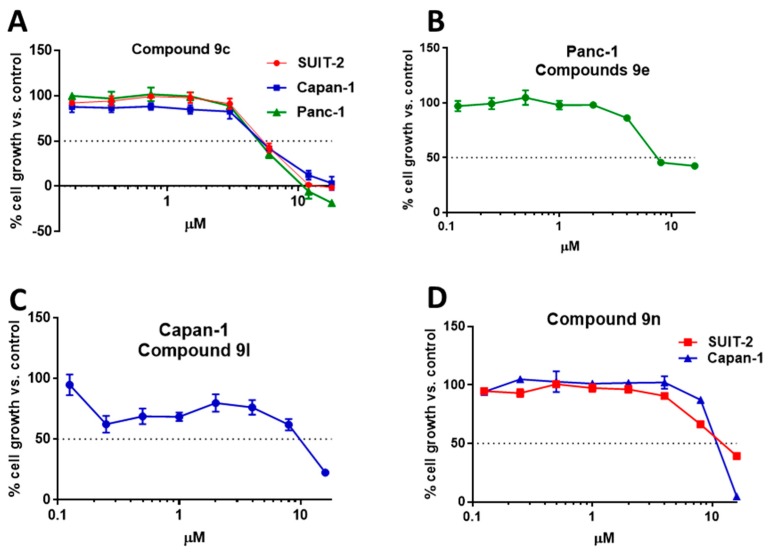
Representative growth inhibition curves of pancreatic ductal adenocarcinoma (PDAC) cells, SUIT-2 (red line), Capan-1 (blue line) and Panc-1 (green line) treated for 72 h with the compound **9c** (**A**), **9e** (**B**), **9l** (**C**) and **9n** (**D**). Points, mean values obtained from three independent experiments; bars, SEM.

The compounds **9c** and **9l** showed relevant antiproliferative activities in all the three preclinical in vitro models, with IC_50s_ ranging from 5.11 to 10.8 µM. Between these two derivatives, the compound **9c** induced the most relevant inhibition of cell growth, showing similar activities in SUIT-2, Capan-1 and Panc-1 cells, with IC_50s_ of 5.5, 5.11 and 5.18 µM, respectively (Figure 2).

The compound **9e** was more active in Panc-1 cells, with IC_50_ value of 10.26 µM, whereas SUIT-2 and Capan-1 cells were resistant to the same treatment and showed IC_50s_ > 16 µM. Conversely, the IC_50_ of the compound **9n** was above 16 µM in Panc-1 cells, while SUIT-2 and Capan-1 cells were more sensitive, with IC_50s_ of 11.8 and 10.49 µM, respectively.

In parallel experiments we evaluated the IC_50_s of the conventional anticancer drugs gemcitabine and 5-fluorouracil, which were below 1 µM. These results were in agreement with previous studies [29,31]. However, because of PDAC chemoresistant nature, these drugs have very limited clinical activity, prompting studies on novel compounds.

Results highlighted that the introduction of a methyl group on the nitrogen atom of the indole moiety was detrimental for the antiproliferative activity against the three cancer cell lines, whereas, substitutions on the phenyl ring as well as the presence of a fluorine atom or a methoxy group on the indole scaffold were not relevant for the activity.

#### 2.2.2. Compound **9c** Inhibited the Migration Rate in SUIT-2, Capan-1 and Panc-1 Cells

**Figure 3:**
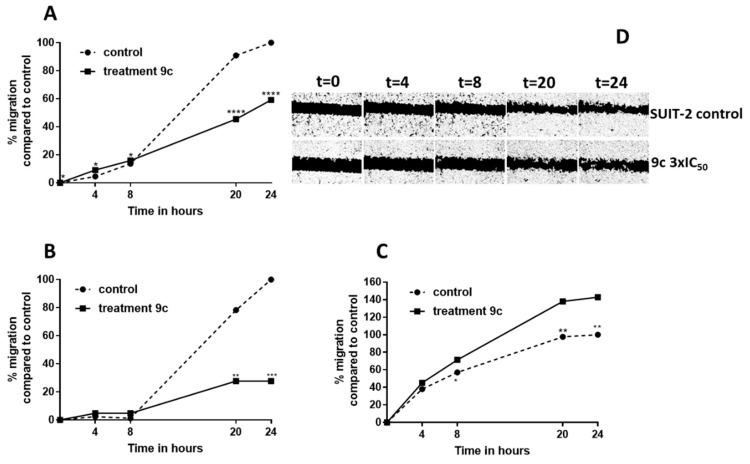
Modulation of migration rate in SUIT-2, Capan-1 and Panc-1 cells treated with imidazo[2,1-*b*][1,3,4]thiadiazole compound **9c**. The percentages of migration were monitored over time at 0, 4, 8, 20, 24 h in SUIT-2 (**A**), Capan-1 (**B**) and Panc-1 (**C**) cells treated with compound **9c** at concentration of 3xIC_50_ values. Points represent the means of at least six different scratch areas. SEM were always below 10%. All the P values were calculated with Student’s-*t*-test. **** *p* < 0.0001, *** *p* < 0.001 ** *p* < 0.01, * *p* < 0.05. (**D**) Representative images of the scratch areas of SUIT-2 cells monitored over time (T = 0 and at 4, 8, 20 and 24 h from the start of the treatment). (*Upper panels*) untreated cells; (*lower panels*) cells treated with compound **9c**. Original magnification 5×.

It is well known that the poor prognosis of PDAC is caused by its early metastatic behaviour. Although many efforts have been made in the multi-omic fields to study the promoters of metastatic events and identify new drug targets, the key drivers of the metastatic properties of PDAC remain unclear [32,33]. However, new therapeutic agents are needed to overcome PDAC aggressiveness and combat PDAC metastasis. Therefore, considering the metastatic nature of SUIT-2, Capan-1 and Panc-1 cells [34,35], we assessed whether or not the most promising compound **9c** was able to affect cell migration. To this goal, the inhibition of cell migration was examined through a high-throughput screening using the scratch wound healing assay, as described previously [36]. SUIT-2, Capan-1 and Panc-1 cells were treated with the compound **9c** at a concentration of 3xIC_50_ and the migration rate was monitored over time within 24 h. Images of the wound closure were taken immediately after scratch (T = 0) and at 4, 8, 20 and 24 h from the treatment. As shown in the Figure 3, the compound **9c** led to a net reduction of the migration in SUIT-2 and Capan-1 cells, whose the percentages of migration compared to the control (set to 100%) were 59.09% and 27.71%, respectively.

Of note these results showed that this compound was able to inhibit migration more than gemcitabine, which in parallel experiments marginally (i.e., less than 15% inhibition) affected PDAC cell migration, as also reported in our previous studies in Panc-1 cells [37]. Conversely, the compound **9c** accelerated migration up to 142.85% in Panc-1 cells. The latter, unexpected results, might be explained by the tendency of PANC-1 cells to clump, a feature which might affect the analysis of the scratch after 24 h. Moreover, though most previous studies, including ours, showed the ability of tyrosine kinase inhibitors (TKIs) to reduce migration in different PDAC cancer cell lines [38], a seminal study on sunitinib and other TKIs reported metastatic acceleration depending on treatment schedule and tumour models [39].

Statistical analyses showed that the above described inhibition of migration in treated cells was significant in all three pre-clinical models, compared to untreated cells. Importantly, to exclude that the wound areas were covered by cell proliferation, we determined the doubling time of each cell lines used, which were above 24 h and we calculated that the area of the wound track which was approximately 10^6^ µm^2^, too large to be covered by only cell proliferation in 24 h, since the average tumor adherent cell surface is around 100–150 µm^2^. Finally, we used concentrations of the compound **9c** that did not induce cell death during 24 h of exposure to the treatments, in fact, we did not observe detached cells after 24-h drug treatment.

## 3. Materials and Methods

### 3.1. Chemistry

All melting points were taken on a Büchi-Tottoly capillary apparatus and are uncorrected. IR spectra were determined in bromoform with a Shimadzu FT/IR 8400S spectrophotometer. ^1^H and ^13^C NMR spectra were measured at 200 and 50.0 MHz, respectively, in DMSO-*d*_6_ solution, using a Bruker Avance II series 200 MHz spectrometer. Column chromatography was performed with Merck silica gel 230–400 mesh ASTM or with Büchi Sepacor chromatography module (prepacked cartridge system). Elemental analyses (C, H, N) were within ±0.4% of theoretical values and were performed with a VARIO EL III elemental analyser. Purity of all the tested compounds was greater than 95%, determined by HPLC (Agilent 1100 Series).

#### 3.1.1. Synthesis of 1*H*-indole-3-carbonitriles (**5a**,**b**)

A solution of the indole **4a**,**b** (5.10 mmol) in anhydrous acetonitrile (4.5 mL) was treated by adding dropwise chlorosulfonyl isocyanate (CSI) (0.44 mL, 5.10 mmol). The reaction mixture was stirred at 0 °C for 2 h, then, anhydrous dimethylformamide (DMF) (2.8 mL, 36.39 mmol) was slowly added and the mixture was stirred at 0 °C for 1.5 h. The resulting solution was poured into crushed ice. The solid obtained was filtered and dried (yields 98%).

Analytical and spectroscopic data for compounds **5a**,**b** are in agreement with those reported in literature [40].

#### 3.1.2. Synthesis of 1-methylindole-3-carbonitriles (**6a**,**b**)

To a solution of the suitable 3-cyanoindole **5a,b** (7.03 mmol) in anhydrous DMF (10 mL) 3.61 mmol of K_2_CO_3_ and dimethyl carbonate (1.8 mL, 21.4 mmol) were added and the mixture was heated at 130 °C for 3.5 h. After cooling (3 °C), water and ice (25 mL) was slowly added under stirring. The suspension obtained was extracted with diethyl ether (3 × 10 mL) and the organic phase was washed with water and brine, was dried over Na_2_SO_4_ and the solvent evaporated at reduced pressure to obtain the 3-cyano-1-methylindole **6a,b** in excellent yields.

Analytical and spectroscopic data are in accordance to those reported in literature [41].

#### 3.1.3. Synthesis of 5-(1*H*-indol-3-yl)-1,3,4-thiadiazol-2-amines (**7a**–**d**)

A mixture of the suitable indole-3-carbonitrile **5a**,**b** or **6a**,**b** (5 mmol), thiosemicarbazide 5mmol) and trifluoroacetic acid (5 mL) was heated at 60 °C for 3.5 h. The reaction mixture was then poured into ice and neutralized with NaHCO_3_ saturated solution. The solid obtained was filtered off, washed with water, cyclohexane and diethyl ether.

##### 5-(5-Methoxy-1H-indol-3-yl)-1,3,4-thiadiazol-2-amine (**7a**)

Orange powder. Yield: 98%, m.p. 216–217 °C, IR cm^−1^: 3604 (NH), 3558 (NH_2_), ^1^H NMR (200 MHz, DMSO-*d*_6_) δ: 3.79 (3H, s, OCH_3_), 6.85–6.91 (1H, m, Ar-H), 7.39 (1H, d, *J* = 8.8 Hz, Ar-H), 7.53 (1H, s, Ar-H), 8.02 (1H, s, Ar-H), 8.58 (2H, bs, NH_2_), 11.80 (1H, s, NH). ^13^C NMR (50 MHz, DMSO-*d*_6_) δ: 55.23 (q), 101.9 (d), 105.7 (s), 112.9 (d), 113.0 (d), 124.3 (s), 128.7 (d), 131.4 (s), 152.1 (s), 154.7 (s), 166.5 (s). Anal. Calcd. for C_11_H_10_N_4_OS (MW: 246.29): C, 53.64%; H, 4.09%; N, 22.75%. Found: C, 53.82%; H, 4.28%; N, 22.53%.

##### 5-(5-Methoxy-1-methyl-1H-indol-3-yl)-1,3,4-thiadiazol-2-amine (**7b**)

Orange powder. Yield: 98%, m.p. 206–207 °C, IR cm^−1^: 3381 (NH_2_), ^1^H NMR (200 MHz, DMSO-*d*_6_) δ: 3.80 (6H, s, OCH_3_, CH_3_), 6.9 (1H, dd, *J* = 2.5, 8.8 Hz, Ar-H), 7.13 (2H, s, NH_2_), 7.41 (1H, d, *J* = 8.9 Hz, Ar-H), 7.61 (1H, d, *J* = 2.3 Hz, Ar-H), 7.81 (1H, s, Ar-H). ^13^C NMR (50 MHz, DMSO-*d*_6_) δ: 32.8 (q), 55.2 (q), 102.3 (d), 106.0 (s), 111.1 (d), 112.5 (d), 124.9 (s), 130.6 (d), 132.0 (s), 152.2 (s), 154.6 (s), 165.4 (s). Anal. Calcd. for C_12_H_12_N_4_OS (MW: 260.31): C, 55.37%; H, 4.65%; N, 21.52%. Found: C, 55.58%; H, 4.81%; N, 21.63%.

##### 5-(5-Fluoro-1H-indol-3-yl)-1,3,4-thiadiazol-2-amine (**7c**)

Orange powder. Yield: 98%, m.p. 257–258 °C IR cm^−1^: 3558 (NH), 3461 (NH_2_), ^1^H NMR (200 MHz, DMSO-*d*_6_) δ: 7.03–7.12 (1H, m, Ar-H), 7.33–7.50 (3H, m, Ar-H, NH_2_), 7.80 (1H, dd, *J* = 2.4, 10.0 Hz, Ar-H), 7.95 (1H, s, Ar-H), 11.79 (1H, s, NH). ^13^C NMR (50 MHz, DMSO-*d*_6_) δ: 105.4 (d, *J_F_* = 24.0 Hz), 107.3 (s), 110.7 (d, *J_F_* = 25.5 Hz), 113.1 (d, *J_F_* = 10.0 Hz), 124.2 (s), 128.5 (d), 133.0 (s), 152.0 (s), 159.9 (s), 165.8 (s). Anal. Calcd. for C_10_H_7_FN_4_S (MW: 234.25): C, 51.27%; H, 3.01%; N, 23.92%. Found: C, 51.42%; H, 3.28%; N, 24.05%.

##### 5-(5-Fluoro-1-methyl-1H-indol-3-yl)-1,3,4-thiadiazol-2-amine (**7d**)

Light orange powder. Yield: 98%, m.p. 183–184 °C IR cm^−1^: 3450 (NH_2_) ^1^H NMR (200 MHz, DMSO-*d*_6_) δ: 3.85 (3H, s, CH_3_), 7.12–7.21 (1H, m, Ar-H), 7.58 (1H, dd, *J* = 2.5, 9.8 Hz, Ar-H), 7.76 (1H, dd, *J* = 2.5, 9.9 Hz, Ar-H), 8.10 (3H, s, Ar-H, NH_2_). ^13^C NMR (50 MHz, DMSO-*d*_6_) δ: 33.1 (q), 105.35 (s), 105.5 (d, *J_F_* = 24.0 Hz), 111.1 (d, *J_F_* = 26.0 Hz), 112.0 (d, *J_F_* = 9.5 Hz), 124.3 (s), 124.5 (s), 133.0 (d), 133.7 (s), 151.3 (s), 155.7 (s). Anal. Calcd. for C_11_H_9_FN_4_S (MW: 248.28): C, 53.21%; H, 3.65%; N, 22.57%. Found: C, 53.50%; H, 3.78%; N, 22.69%.

#### 3.1.4. Synthesis of 3-(6-phenylimidazo[2,1-*b*][1,3,4]thiadiazol-2-yl)-1H-indole hydrobromides (**9a**–**p**)

A mixture of 5-(1*H*-indol-3-yl)-1,3,4-thiadiazol-2-amine **7a**–**d** (0.92 mmol) and the 2-bromoethanone **8a**–**f** (0.92 mmol) in 40 mL of anhydrous ethanol was stirred at reflux for 24 h. After cooling at room temperature, the obtained solid was filtered off and washed with cold ethanol. Derivative **9p** was characterized only by ^1^H NMR spectra due to its poor solubility.

##### 5-Methoxy-3-(6-phenylimidazo[2,1-b][1,3,4]thiadiazol-2-yl)-1H-indole hydrobromide **9a**

Light brown solid. Yield: 80%, m.p. 234–235 °C. IR cm^−1^: 3592 (NH), 3433 (NH); ^1^H NMR (200 MHz, DMSO-*d*_6_) δ: 3.85 (3H, s, OCH_3_), 6.94 (1H, dd, *J* = 2.2, 8.7 Hz, Ar-H), 7.32–7.49 (4H, m, Ar-H), 7.63 (1H, s, Ar-H), 7.88 (2H, d, *J* = 7.6 Hz, Ar-H), 8.33 (1H, d, *J* = 7.6 Hz, Ar-H), 8.84 (1H, s, Ar-H), 12.10 (1H, s, NH). ^13^C NMR (50 MHz, DMSO-*d*_6_) δ: 55.3 (q), 102.2 (d), 105.8 (s), 110.9 (d), 113.1 (d), 113.4 (d), 124.3 (s), 124.6 (2xd), 127.7 (d), 128.8 (2xd), 130.2 (d), 131.6 (s), 132.3 (s), 142.6 (s), 142.7 (s), 155.2 (s), 158.3 (s). Anal. Calcd. for C_19_H_15_BrN_4_OS (MW: 427.3): C, 53.40%; H, 3.54%; N, 13.11%. Found: C, 53.62%; H, 3.70%; N, 13.05%.

##### 5-Methoxy-3-[6-(4-fluorophenyl)imidazo[2,1-b][1,3,4]thiadiazol-2-yl]-5-methoxy-1H-indole hydrobromide **9b**

Light grey solid. Yield: 75%, m.p. 277–278 °C. IR cm^−1^: 3615 (NH), 3392 (NH); ^1^H NMR (200 MHz, DMSO-*d*_6_) δ: 3.85 (3H, s, OCH_3_), 6.48 (1H, bs, NH), 6.92–6.96 (2H, d, *J* = 8.7 Hz, Ar-H), 7.24–7.33 (2H, d, *J* = 2.3 Hz, Ar-H), 7.43–7.44 (1H, d, *J* = 8.8 Hz, Ar-H), 7.62 (1H, s, Ar-H), 7.88–7.94 (2H, m, Ar-H), 8.29–8.31 (1H, d, *J* = 2.29 Hz, Ar-H), 8.77 (1H, s, Ar-H), 12.08 (1H, bs, NH). ^13^C NMR (50 MHz, DMSO-*d*_6_) δ: 55.3 (q), 102.2 (d), 106.6 (s), 110.6 (d), 113.1 (d), 113.3 (d), 115.4 (d), 115.8 (d), 124.3 (s), 126.4 (d), 126.6 (2xd), 129.5 (s), 130.0 (s), 131.6 (s), 142.4 (s), 142.8 (s), 155.1 (s), 157.9 (s). Anal. Calcd. for C_19_H_14_BrFN_4_OS (MW: 445.31): C, 51.25%; H, 3.17%; N, 12.58%. Found: C, 51.45%; H, 3.33%; N, 12.65%.

##### 5-Methoxy-3-[6-(4-nitrophenyl)imidazo[2,1-b][1,3,4]thiadiazol-2-yl]-1H-indole **9c**

Dark yellow solid. Yield: 73%, m.p. 291–292 °C. IR cm^−1^: 3609 (NH); ^1^H NMR (200 MHz, DMSO-*d*_6_) δ: 3.86 (3H, s, OCH_3_), 6.94 (1H, dd, *J* = 2.3, 8.8, Ar-H), 7.41–7.45 (1H, d, *J* = 8.8 Hz, Ar-H), 7.62 (1H, d, *J* = 2.2 Hz, Ar-H), 8.06 (1H, s, Ar-H), 8.11 (1H, s, Ar-H), 8.21 (1H, s, Ar-H), 8.25 (2H, d, *J* = 2.2, Ar-H), 8.96 (1H, s, Ar-H), 12.03 (1H, bs, NH). ^13^C NMR (50 MHz, DMSO-*d*_6_) δ: 55.3 (q), 102.2 (d), 106.2 (s), 113.0 (d), 113.3 (d), 124.1 (3xd), 124.3 (s), 124.8 (2xd), 130.0 (d), 131.6 (s), 140.6 (s), 142.4 (s), 144.0 (s), 145.6 (s), 155.1 (s), 158.1 (s). Anal. Calcd. for C_19_H_13_N_5_O_3_S (MW: 391.40): C, 58.30%; H, 3.35%; N, 17.89%. Found: C, 58.46%; H, 3.48%; N, 18.02%.

##### 5-Methoxy-3-[6-(3-methoxyphenyl)imidazo[2,1-b][1,3,4]thiadiazol-2-yl]-1H-indole hydrobromide **9d**

Light brown solid. Yield: 78%, m.p. 272–273 °C. IR cm^−1^: 3604 (NH), 3142 (NH); ^1^H NMR (200 MHz, DMSO-*d*_6_) δ: 3.83 (3H, s, OCH_3_), 3.85 (3H, s, OCH_3_), 6.87–6.98 (2H, m, Ar-H), 7.32–7.48 (4H, m, Ar-H), 7.63 (1H, d, *J* = 2.28 Hz, Ar-H), 8.33 (1H, d, *J* = 2.9 Hz, Ar-H), 8.61 (1H, s, NH), 8.86 (1H, s, Ar-H), 12.09 (1H, bs, NH). ^13^C NMR (50 MHz, DMSO-*d*_6_) δ: 55.1 (q), 55.3 (q), 102.1 (d), 106.0 (s), 109.8 (2xd), 111.1 (d), 113.1 (s), 113.4 (2xd), 116.9 (d), 124.3 (s), 129.9 (2xd), 131.6 (s), 134.2 (s), 140.3 (s), 155.1 (s), 159.3 (s), 159.6 (s). Anal. Calcd. for C_20_H_17_BrN_4_O_2_S (MW: 457.34): C, 52.52%; H, 3.75%; N, 12.25%. Found: C, 52.69%; H, 3.82%; N, 12.36%.

##### 5-Methoxy-3-[6-(2,5-dimethoxyphenyl)imidazo[2,1-b][1,3,4]thiadiazol-2-yl]-5-methoxy-1H-indole **9e**

Yellow solid. Yield: 78%, m.p. 261–262 °C. IR cm^−1^: 3604 (NH); ^1^H NMR (200 MHz, DMSO-*d*_6_) δ: 3.78 (3H, s, OCH_3_), 3.85 (3H, s, OCH_3_), 3.94 (3H, s, OCH_3_), 6.88–6.96 (2H, m, Ar-H), 7.07 (1H, d, *J* = 9.0 Hz, Ar-H), 7.43–7.65 (3H, m, Ar-H), 8.36 (1H, d, *J* = 3.0 Hz, Ar-H), 8.71 (1H, s, NH), 12.12 (1H, bs, NH). ^13^C NMR (50 MHz, DMSO-*d*_6_) δ: 55.4 (2xq), 55.9 (q), 102.3 (d), 105.7 (s), 111.6 (3xd), 112.8 (s), 113.1 (d), 113.4 (d), 113.6 (d) 124.3 (s), 130.5 (d), 131.6 (s), 137.3 (s), 141.8 (s), 149.8 (s), 153.2 (s), 155.2 (s), 158.8 (s). Anal. Calcd. for C_21_H_18_N_4_O_3_S (MW: 406.57): C, 62.05%; H, 4.46%; N, 13.78%. Found: C, 62.58%; H, 4.58%; N, 13.86%.

##### 5-Methoxy-3-{6-[4-(trifluoromethyl)phenyl]imidazo[2,1-b][1,3,4]thiadiazol-2-yl}-1H-indole hydrobromide **9f**

White solid. Yield: 68%, m.p. 198–199 °C. IR cm^−1^: 3609 (NH), 3228 (NH); ^1^H NMR (200 MHz, DMSO-*d*_6_) δ: 3.86 (3H, s, OCH_3_), 6.43 (1H, bs, NH), 6.92 (1H, d, *J* = 8.8 Hz, Ar-H), 7.45 (1H, dd, *J* = 2.3, 8.8 Ar-H), 7.64 (1H, s, Ar-H), 7.77 (2H, d, *J* = 7.5, Hz, Ar-H), 8.09 (2H, d, *J* = 7.7, Hz, Ar-H), 8.28 (1H, s, Ar-H), 8.91 (1H, s, Ar-H), 12.06 (1H, bs, NH). ^13^C NMR (50 MHz, DMSO-d6) δ: 55.3 (q), 102.2 (d), 106.1 (s), 112.1 (d), 113.0 (d), 113.3 (d), 124.3 (s), 124.8 (3xd) 125.6 (d), 130.0 (d), 131.6 (2xs), 137.8 (s), 142.7 (s), 143.5 (s), 155.1 (2xs), 157.9 (s). Anal. Calcd. for C_20_H_14_BrF_3_N_4_OS (MW: 495.31): C, 48.50%; H, 2.85%; N, 11.31%. Found: C, 48.69%; H, 2.98%; N, 11.26%.

##### 5-Methoxy-1-methyl-3-(6-phenylimidazo[2,1-b][1,3,4]thiadiazol-2-yl)-1H-indole hydrobromide **9g**

Light grey solid. Yield: 81%, m.p. 252–253 °C. IR cm^−1^: 3592 (NH); ^1^H NMR (200 MHz, DMSO-*d*_6_) δ: 3.85 (6H, s, OCH_3_, CH_3_), 6.99 (1H, dd, *J* = 2.4, 8.9 Hz, Ar-H), 7.28–7.60 (5H, m, Ar-H), 7.84 (2H, d, *J* = 7.1 Hz, Ar-H), 8.33 (1H, s, Ar-H), 8.52 (1H, bs, NH), 8.81 (1H, s, Ar-H). ^13^C NMR (50 MHz, DMSO-*d*_6_) δ: 33.3 (q, CH_3_), 55.3 (q, OCH_3_), 102.4 (d), 104.5 (s), 110.9 (d), 111.9 (s), 112.9 (d), 124.6 (2xd), 127.7 (d) 128.8 (2xd), 132.1 (s), 132.3 (s), 133.6 (d), 142.6 (s), 142.5 (s), 155.5 (s), 157.8 (s). Anal. Calcd. for C_20_H_17_BrN_4_OS (MW: 441.34): C, 54.43%; H, 3.88%; N, 12.69%. Found: C, 54.59%; H, 4.00%; N, 12.96%.

##### 5-Methoxy-3-[6-(4-fluorophenyl)imidazo[2,1-b][1,3,4]thiadiazol-2-yl]-5-methoxy-1-methyl-1H-indole hydrobromide **9h**

Light yellow solid. Yield: 72%, m.p. 278–279 °C. IR cm^−1^: 3609 (NH); ^1^H NMR (200 MHz, DMSO-*d*_6_) δ: 3.84 (6H, s, OCH_3_, CH_3_), 6.99 (1H, dd, *J* = 2.4, 8.9 Hz, Ar-H), 7.22–7.31 (2H, m, Ar-H), 7.48–7.52 (1H, d, *J* = 8.9 Hz, Ar-H), 7.58 (1H, d, *J* = 2.0 Hz, Ar-H), 7.95–7.82 (2H, m, Ar-H), 8.29 (1H, s, Ar-H), 8.74 (1H, s, Ar-H), 9.66 (1H, bs, NH). ^13^C NMR (50 MHz, DMSO-*d*_6_) δ: 33.2 (q), 55.4 (q), 102.3 (d), 104.7 (s), 110.6 (d), 111.9 (d), 112.9 (d), 115.4 (d), 115.8 (d), 124.6 (s), 126.4 (d) 126.5 (d), 129.6 (s), 132.3 (s), 133.4 (d), 142.6 (2xs), 155.4 (2xs), 157.3 (s). Anal. Calcd. for C_20_H_16_BrN_4_OS (MW: 459.33): C, 52.30%; H, 3.51%; N, 12.20%. Found: C, 52.50%; H, 4.67%N, 12.35%.

##### 5-Methoxy-1-methyl-3-[6-(3-methoxyphenyl)imidazo[2,1-b][1,3,4]thiadiazol-2-yl]-1H-indole hydrobromide **9i**

Brown solid. Yield: 60%, m.p. 260–261 °C. IR cm^−1^: 3461 (NH); ^1^H NMR (200 MHz, DMSO-*d*_6_) δ: 3.82 (3H, s, CH_3_), 3.85 (6H, s, OCH_3_), 5.99 (1H, bs, NH), 6.86 (H, d, *J* = 7.3 Hz, Ar-H), 6.99 (1H, dd, *J* = 2.4, 8.9 Hz, Ar-H), 7.30–7.61 (5H, m, Ar-H), 8.32 (1H, s, Ar-H), 8.81 (1H, s, Ar-H). ^13^C NMR (50 MHz, DMSO-*d*_6_) δ: 33.3 (q), 55.0 (q), 55.3 (q, OCH_3_), 102.3 (d), 104.7 (s), 109.7 (2xd), 111.9 (d), 112.9 (d), 113.2 (s), 116.8 (d), 124.6 (s), 129.8 (2xd), 130.5 (d), 132.3 (s), 134.0 (s), 142.5 (s), 142.9 (s), 155.4 (s), 157.6 (s), 159.6 (s). Anal. Calcd. for C_21_H_19_BrN_4_O_2_S (MW: 471.46): C, 53.51%; H, 4.06%; N, 11.89%. Found: C, 53.68%; H, 4.15%; N, 11.99%.

##### 5-Methoxy-3-[6-(2,5-dimethoxyphenyl)imidazo[2,1-b][1,3,4]thiadiazol-2-yl]-5-methoxy-1-methyl-1H-indole **9j**

White solid. Yield: 65%, m.p. 277–278 °C. ^1^H NMR (200 MHz, DMSO-*d*_6_) δ: 3.77 (6H, s, CH_3_), 3.86 (6H, s, CH_3_), 6.86–7.09 (3H, m, Ar-H), 7.51 (2H, dd, *J* = 2.4, 8.9 Hz, Ar-H), 7.59–7.66 (2H, m, Ar-H); 8.34 (1H, s, Ar-H). ^13^C NMR (50 MHz, DMSO-*d*_6_) δ: 33.3 (q), 55.2 (q), 55.4 (q), 55.9 (q), 99.4 (s), 102.4 (d), 104.4 (s), 111.5 (2xd), 112.6 (s), 112.8 (2xd), 112.9 (d), 114.2 (d), 124.6 (s), 132.3 (2xs), 133.8 (d), 141.6 (s), 149.8 (s), 153.1 (s), 155.5 (s). Anal. Calcd. for C_22_H_10_BrN_4_O_3_S (MW: 420.48): C, 62.84%; H, 4.79%; N, 13.32%. Found: C, 62.95%; H, 4.89%; N, 13.39%.

##### 5-Methoxy-1-methyl-3-{6-[4-(trifluoromethyl)phenyl]imidazo[2,1-b][1,3,4]thiadiazol-2-yl}-1H-indole **9k**

Light grey solid. Yield: 67%, m.p. 216 °C. ^1^H NMR (200 MHz, DMSO-*d*_6_) δ: 3.85(6H, s, CH_3_, OCH_3_), 6.98 (1H, dd, *J* = 2.4, 8.9Hz, Ar-H), 7.48 (1H, d, *J* = 8.9 Hz, Ar-H), 7.61 (1H, d, *J* = 2.3 Hz, Ar-H), 7.74 (2H, d, *J* = 8.0, Hz, Ar-H), 8.06 (2H, d, *J* = 8.1, Hz, Ar-H), 8.24 (1H, s, Ar-H), 8.85 (1H, s, Ar-H). ^13^C NMR (50 MHz, DMSO-*d*_6_) δ: 33.2 (q), 55.3 (q), 99.4 (s), 102.4 (d), 104.9 (2xs), 111.9 (d), 112.1 (s), 112.9 (d), 124.7 (4xd), 125.5 (d), 132.3 (s), 133.2 (d), 138.0 (s), 143.0 (s), 155.4 (2xs), 157.3 (s). Anal. Calcd. for C_21_H_15_F_3_N_4_OS (MW: 428.43): C, 58.87%; H, 3.53%; N, 13.08%. Found: C, 59.01%; H, 3.62%; N, 13.14%.

##### 5-Fluoro-3-(6-phenylimidazo[2,1-b][1,3,4]thiadiazol-2-yl)-1H-indole hydrobromide **9l**

White solid. Yield: 72%, m.p. 278–279 °C. IR cm^−1^: 3145 (NH) 2890 (NH).; ^1^H NMR (200 MHz, DMSO-*d*_6_) δ: 7.03 (1H, bs, NH), 7.15 (1H, td, *J* = 2.5, 10.1, 9.6 Hz, Ar-H) 7.26–7.47(3H, m, Ar-H), 7.57 (1H, dd, *J* = 4.6, 9.0 Hz Ar-H), 7.82–7.90 (3H, m, Ar-H), 8.41 (1H, d, Ar-H), 8.75 (1H, s, Ar-H) 12.28 (1H, bs, NH). ^13^C NMR (50 MHz, DMSO-d6) δ: 105.0 (d), 106.5 (s), 110.0 (d), 110.6 (s), 111.2 (d), 111.7 (d), 123.9 (s), 124.5 (2xd), 127.3 (d), 128.7 (2xd), 131.3 (d), 133.3 (2xs), 142.8 (s), 143.9 (s), 157.2 (s). Anal. Calcd. for C_18_H_12_BrFN_4_S (MW: 415.28): C, 52.06%; H, 2.91%; N, 13.49%. Found: C, 52.16%; H, 3.02%; N, 13.59%.

##### 5-Fluoro-3-[6-(3-methoxyphenyl)imidazo[2,1-b][1,3,4]thiadiazol-2-yl]-1H-indole hydrobromide **9m**

White solid. Yield: 74%, m.p. 282–283 °C. IR cm^−1^: 3153 (NH), 3658 (NH); ^1^H NMR (200 MHz, DMSO-*d*_6_) δ: 3.82 (3H, s, OCH_3_), 6.87–7.62 (6H, m, Ar-H), 7.83 (1H, dd, *J* = 2.3, 9.7 Hz, Ar-H), 8.44 (1H, d, *J* = 3 Hz, Ar-H), 8.81 (1H, s, Ar-H), 9.01 (1H, bs, NH), 12.30 (1H, bs, NH). ^13^C NMR (50 MHz, DMSO-*d*_6_) δ: 55.0 (q), 99.4 (s), 105.0 (d), 109.8 (2xd), 110.4 (s), 111.0 (s), 111.3 (d), 113.2 (d), 113.8 (s), 114.0 (d), 116.9 (d), 118.7 (s), 124.1 (s), 129.8 (d), 131.4 (d), 133.3 (s), 142.7 (s), 143.2 (s), 151.6 (2xs). Anal. Calcd. for C_19_H_14_BrFN_4_OS (MW: 445.30): C, 51.25%; H, 3.17%; N, 12.58%. Found: C, 51.39%; H, 3.25%; N, 12.64%.

##### 5-Fluoro-3-{6-[4-(trifluoromethyl)phenyl]imidazo[2,1-b][1,3,4]thiadiazol-2-yl}-1H-indole hydrobromide **9n**

White solid. Yield: 68%, m.p. 259–260 °C. IR cm^−1^: 3155 (NH), 2925 (NH); ^1^H NMR (200 MHz, DMSO-*d*_6_) δ: 6.16 (1H, bs, NH), 7.15 (1H, td, *J* = 2.5, 9.1, 9.0 Hz, Ar-H), 7.56 (1H, dd, *J* = 4.5, 8.5 Hz, Ar-H), 7.73–7.86 (3H, m, Ar-H), 8.05 (2H, d, *J* = 8.1 Hz, Ar-H), 8.41 (1H, d, *J* = 2.9 Hz, Ar-H), 8.88 (1H, s, Ar-H), 12.27 (1H, bs, NH). ^13^C NMR (50 MHz, DMSO-*d*_6_) δ: 105.6 (d), 112.1 (d), 113.2 (d), 113.9 (d), 114.0 (s), 123.9 (s), 124.1 (s), 124.7 (4xd), 125.6 (d), 126.7 (s), 131.3 (d), 133.3 (s), 137.9(s), 143.0 (s), 143.5 (s), 157.4 (s). Anal. Calcd. for C_19_H_11_BrF_4_N_4_S (MW: 483.27): C, 47.22%; H, 2.29%; N, 11.59%. Found: C, 47.36%; H, 2.36%; N, 11.65%.

##### 5-Fluoro-3-[6-(2,5-dimethoxyphenyl)imidazo[2,1-b][1,3,4]thiadiazol-2-yl]-1H-indole hydrobromide **9o**

White solid. Yield: 76%, m.p. 266 °C. IR cm^−1^: 3427 (NH), 3093 (NH); ^1^H NMR (200 MHz, DMSO-*d*_6_) δ: 3.77 (3H, s, CH_3_), 3.92 (3H, s, CH_3_), 4.25 (1H, bs, NH), 6.85 (1H, dd, *J* = 2.9, 8.8 Hz, Ar-H), 7.02–7.20 (2H, m, 2xAr-H), 7.56 (1H, dd, *J* = 4.3, 8.5 Hz, Ar-H), 7.70 (1H, d, *J* = 2.86 Hz, Ar-H), 7.87 (1H, d, *J* = 89.6 Hz, Ar-H), 8.41 (1H, s, Ar-H), 8.55 (1H, s, Ar-H), 12.24 (1H, bs, NH). ^13^C NMR (50 MHz, DMSO-d6) δ: 55.3 (q), 55.7 (q), 105.1 (d), 105.6 (d), 106.6 (s), 111.5 (2xd), 112.5 (2xd), 113.8 (d), 122.2 (s), 124.0 (s), 131.1 (d), 133.3 (s), 139.7 (s), 142.0 (s), 149.8 (s), 153.1 (s), 156.9 (s). Anal. Calcd. for C_20_H_16_BrFN_4_O_2_S (MW: 475.33): C, 50.54%; H, 3.39%; N, 11.79%. Found: C, 50.68%; H, 3.52%; N, 11.86%.

##### 5-Fluoro-1-methyl-3-{6-[4-(trifluoromethyl)phenyl]imidazo[2,1-b][1,3,4]thiadiazol-2-yl}-1H-indole hydrobromide **9p**

Grey solid. Yield: 78%, m.p. 281 °C. IR cm^−1^: 3501 (NH); ^1^H NMR (200 MHz, DMSO-*d*_6_) δ: 3.85 (3H, s, CH_3_), 7.22 (1H, td, *J* = 2.6, 9.1, 9.1 Hz, Ar-H), 7.57–7.81 (5H, m, Ar-H, NH), 8.02 (2H, d, *J* = 8.0 Hz, Ar-H), 8.36 (1H, s, Ar-H), 8.81 (1H, s, Ar-H). Anal. Calcd. for C_20_H_13_BrF_4_N_4_S (MW: 497.30): C, 48.30%; H, 2.63%; N, 11.27%. Found: C, 48.50%; H, 2.75%; N, 11.36%.

### 3.2. Biology

#### 3.2.1. Drugs and Chemical

The 3-(6-phenylimidazo[2,1-*b*][1,3,4]thiadiazol-2-yl)-1*H-*indole compounds **9a**–**p** were synthesised at the Department of Pharmacy, University of Palermo (Palermo, Italy). The drugs were dissolved in DMSO. The medium, foetal bovine serum (FBS), penicillin (50 IU mL^−1^) and streptomycin (50 µg mL^−1^) were from Gibco (Gaithersburg, MD, USA). All other chemicals were from Sigma (Zwijndrecht, the Netherlands).

#### 3.2.2. Cell Cultures

Capan-1 and Panc-1 cell lines were purchased at the ATCC (American Type Culture Collection) (Manassas, VA, USA), while SUIT-2 cells were a generous gift from Dr. Adam Frampton (Imperial College, London, UK). The cell lines were tested for their authentication by STR–PCR (Short Tandem Repeat- Polymerase Chain Reaction), performed by BaseClear (Leiden, the Netherlands). The cells were cultured in RPMI-1640 (Roswell Park Memorial Institute 1640) supplemented with 10% heat-inactivated FBS, 1% penicillin/streptomycin or in DMEM (Dulbecco’s Modified Eagle’s Medium), supplemented with 10% heat-inactivated FBS, 1% HEPES (4-(2-hydroxyethyl)-*1*-piperazineethanesulfonic acid). The cells were kept in a humidified atmosphere of 5% CO_2_ and 95% air at 37 °C and harvested with trypsin-EDTA (Ethylenediaminetetraacetic acid).

#### 3.2.3. Cell Growth Inhibition

To evaluate the inhibitory effects of the imidazo[2,1-*b*][1,3,4]thiadiazole compounds **9a**–**p** on cell growth, we performed the Sulforhodamine-B (SRB) assay, as previously described [42]. Cells were seeded into 96-well flat-bottom plates in triplicate at a density of 3 × 10^3^ cells/well for SUIT-2 and Panc-1, while 5 × 10^3^ cells/well were used for Capan-1. Cells were incubated at 37 °C for 24 h to create a confluent monolayer and then they were treated with 100 µL of increasing concentrations of the compounds dissolved in DMSO.

After 72 h of treatment, the cells were fixed with 25 µL of 50% cold trichloroacetic acid (TCA) and kept for at least 60 min at 4 °C. Then, the plates were washed gently with deionized water, dried at room temperature (RT) overnight and stained with 50 µL of 0.4% SRB solution in 1% acetic acid for 15 min at RT. The excess of SRB stain was removed on dried tissues and the plates were washed with 1% acetic acid and let dry at RT overnight. The SRB was dissolved in 150 µL of tris(hydroxymethyl)aminomethane solution pH = 8.8 (TRIS base) and the optical density (OD) was detected at a wavelength of 490 nm and 540 nm. Cell growth inhibition was calculated as the percentage versus vehicle-treated cells (“negative control”) OD (corrected for OD before drug addiction). Finally, the half maximal inhibitory concentration (IC_50_) was calculated by non-linear least squares curve fitting (GraphPad Prism 7, Intuitive Software for Science, San Diego, CA, USA).

#### 3.2.4. Wound-Healing Assays

The in vitro scratch wound-healing assay was performed as previously described [43]. SUIT-2, Capan-1 and Panc-1 cells were seeded into a 96-well plates at a density of 5 × 10^4^ cells/well and incubated for 24 h at 37 °C, 5% CO_2_ and 100% humidity. Then, cell monolayer was scratched through a specific needle to create a scratch of constant width. After removal of the detached cells by washing with phosphate buffered saline (PBS), we added only medium in the control wells and medium with the compounds of interest in the experimental wells. The wound closure was monitored by phase-contrast microscopy using a Universal Grab 6.3 software (Digital Cell Imaging Labs, Keerbergen, Belgium) integrated to the Leica DMI300B migration station (Leica Microsystems, Eindhoven, Netherlands) and the pictures were captured immediately after scratch (T = 0) and at 4, 8, 20 and 24 h from the treatment. The results were analysed with the Scratch Assay 6.2 software (Digital Cell Imaging Labs).

#### 3.2.5. Statistical Analysis

All SRB assays were carried out in triplicate and repeated at least three times, whereas the percentages of cell migration were calculated taking into account at least six scratch areas. The data were evaluated using GraphPad Prism (GraphPad Software, San Diego, CA, USA). Data were expressed as mean values ± SEM and analysed by the Student *t* test.

## 4. Conclusions

PDAC is one of the deadliest cancer types and despite enormous efforts in pancreatic cancer research, in 2019, the American Cancer Society estimated 1,762,450 new cancer cases and 606,880 cancer deaths in the United States (US) [44]. Due to the lack of clinical signs and symptoms, most patients are diagnosed at an advanced/unresectable stage of the disease and regimens with combinations of conventional chemotherapy drugs are the best option for the treatment of PDAC patients. [45]. However, PDACs are characterized by common inherent or acquired resistance to conventional treatment modalities and new therapeutic strategies are warranted [33,46]. A new series of imidazo[2,1-*b*][1,3,4]thiadiazole derivatives **9a**–**p** were efficiently synthesized and tested for their in vitro antiproliferative properties on a panel of PDAC cell lines, including SUIT-2, Capan-1 and Panc-1. Four out of sixteen compounds (**9c**, **9e**, **9l** and **9n**), showed interesting in vitro antiproliferative activity. In particular, the compounds **9c** and **9l** were active in all three preclinical models with IC_50s_ ranging from 5.1 to 10.8 µM. Notably, the IC_50s_ of the compound **9c** in SUIT-2, Capan-1 and Panc-1 cells were the lowest (around 5 µM). The compound **9e** was active only in Panc-1 cells. Conversely, the compound **9n** inhibited cell proliferation in SUIT-2 and Capan-1 cells, with IC_50s_ of 11.8 ± 0.54 and 10.49 ± 0.16 µM, respectively. Finally, using the scratch wound-healing assay, we demonstrated a relevant anti-migratory activity of the compound **9c** in SUIT-2 and Capan-1 cells. Overall, the results of cytotoxicity and cell migration obtained with compound **9c** could suggest its role as an interesting hit compound to create a library of new derivatives and study in deep the structure–activity relationship (SAR). This could indeed be extremely useful to guide the synthesis of future analogues. In particular, we focused our research on derivatives bearing at position 5 of indole scaffold a group (-OCH3) or atom (-F) with electron-withdrawing properties. Furthermore, we investigated how the N-methyl indole, as well as the effect of different substitutions on the phenyl ring, could influence activity. As reported in the Table 1, some of the compounds 1*H*-indole showed antiproliferative activity in one or more cell lines, unlike the 1-methyl-1*H*-indole analogues. Probably, this is due to the ability to create hydrogen bonds with the target. Concerning the substitution on phenyl ring, we did not observe differences between the presence or absence of electron-withdrawing groups, with the exception of nitro group (-NO_2_) that, thanks to the delocalization of negative and positive charges, increased the cytotoxicity activity in all the cell lines.

## Figures and Tables

**Table 1:**
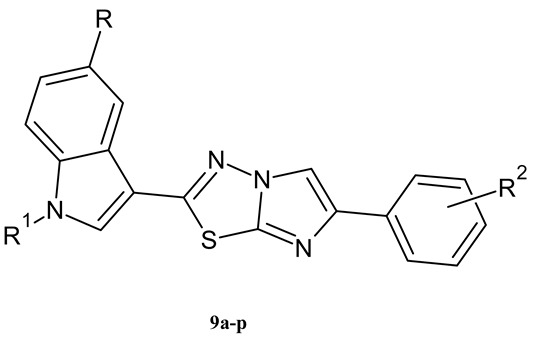
Chemical structure of compounds **9a–p**.

Compound	R	R1	R2	Yield (%)
**9a**	OCH_3_	H	H	80%
**9b**	OCH_3_	H	4-F	75%
**9c**	OCH_3_	H	4-NO_2_	73%
**9d**	OCH_3_	H	3-OCH_3_	78%
**9e**	OCH_3_	H	2,5-OCH_3_	78%
**9f**	OCH_3_	H	4-CF_3_	68%
**9g**	OCH_3_	CH_3_	H	81%
**9h**	OCH_3_	CH_3_	4-F	72%
**9i**	OCH_3_	CH_3_	3-OCH_3_	60%
**9j**	OCH_3_	CH_3_	2,5-OCH_3_	65%
**9k**	OCH_3_	CH_3_	4-CF_3_	67%
**9l**	F	H	H	72%
**9m**	F	H	3-OCH_3_	74%
**9n**	F	H	4-CF_3_	68%
**9o**	F	H	2,5-OCH_3_	76%
**9p**	F	CH_3_	4-CF_3_	78%

**Table 2:** IC_50s_ of SUIT-2, Capan-1 and Panc-1 cells treated with compounds **9a–p**.

IC_50_^a^ (µM) ± SEM ^b^ Cell Lines			
Comp	SUIT-2	Capan-1	Panc-1
**9a**	>16	>16	>16
**9b**	>16	>16	>16
**9c**	5.5 ± 0.19	5.11 ± 0.29	5.18 ± 0.12
**9d**	>16	>16	>16
**9e**	>16	>16	10.26 ± 0.20
**9f**	>16	>16	>16
**9g**	>16	>16	>16
**9h**	>16	>16	>16
**9i**	>16	>16	>16
**9j**	>16	>16	>16
**9k**	>16	>16	>16
**9l**	10.4 ± 0.07	8.57 ± 0.51	10.8 ± 0.13
**9m**	>16	>16	>16
**9n**	11.8 ± 0.54	10.49 ±0.16	>16
**9o**	>16	>16	>16
**9p**	>16	>16	>16

^a^ The values are reported as means ± SEM of three separated experiments. ^b^ SEM^:^ Standard Error Mean.

## References

[1] Torre L.A., Siegel R.L., Ward E.M., Jemal A. (2016). Global Cancer Incidence and Mortality Rates and Trends—An Update. Cancer Epidemiol. Biomark. Prev..

[2] Sung H., Siegel R.L., Rosenberg P.S., Jemal A. (2019). Emerging cancer trends among young adults in the USA: Analysis of a population-based cancer registry. Lancet Public Health.

[3] Jadhav V.B., Kulkarni M.V., Rasal V.P., Biradar S.S., Vinay M.D. (2008). Synthesis and anti-inflammatory evaluation of methylene bridged benzofuranyl imidazo[2,1-*b*][1,3,4] thiadiazoles. Eur. J. Med. Chem..

[4] Agatonovic-Kustrin S., Kettle C., Morton D.W. (2018). A molecular approach in drug development for Alzheimer’s disease. Biomed. Pharmacother..

[5] Tahghighi A., Razmi S., Mahdavi M., Foroumadi P., Ardestani S.K., Emami S., Kobarfard F., Dastmalchi S., Shafiee A., Foroumadi A. (2012). Synthesis and anti-leishmanial activity of 5-(5-nitrofuran-2-yl)-1,3,4-thiadiazol-2-amines containing N-[(1-benzyl-1H-1,2,3-triazol-4-yl) methyl] moieties. Eur. J. Med. Chem..

[6] Jakovljević K., Matić I.Z., Stanojković T., Krivokuća A., Marković V., Joksović M.D., Mihailović N., Nićiforović M., Joksović L. (2017). Synthesis, antioxidant and antiproliferative activities of 1,3,4-thiadiazoles derived from phenolic acids. Bioorg. Med. Chem. Lett..

[7] Alegaon S.G., Alagawadi K.R., Sonkusare P.V., Chaudhary S.M., Dadwe D.H., Shah A.S. (2012). Novel imidazo[2,1-*b*] [1,3,4] thiadiazole carrying rhodanine-3-acetic acid as potential antitubercular agents. Bioorg. Med. Chem. Lett..

[8] Bhongade B.A., Talath S., Gadad R.A., Gadad A.K. (2016). Biological activities of imidazo[2,1-*b*] [1,3,4] thiadiazole derivatives: A review. J. Saudi Chem. Soc..

[9] Schillaci D., Spanò V., Parrino B., Carbone A., Montalbano A., Barraja P., Diana P., Cirrincione G., Cascioferro S. (2017). Pharmaceutical Approaches to Target Antibiotic Resistance Mechanisms. J. Med. Chem..

[10] Cascioferro S., Parrino B., Petri G.L., Cusimano M.G., Schillaci D., Di Sarno V., Musella S., Giovannetti E., Cirrincione G., Diana P. (2019). 2,6-Disubstituted imidazo[2,1-*b*] [1,3,4] thiadiazole derivatives as potent staphylococcal biofilm inhibitors. Eur. J. Med. Chem..

[11] Parrino B., Schillaci D., Carnevale I., Giovannetti E., Diana P., Cirrincione G., Cascioferro S. (2019). Synthetic small molecules as anti-biofilm agents in the struggle against antibiotic resistance. Eur. J. Med. Chem..

[12] Karki S.S., Panjamurthy K., Kumar S., Nambiar M., Ramareddy S.A., Chiruvella K.K., Raghavan S.C. (2011). Synthesis and biological evaluation of novel 2-aralkyl-5-substituted-6-(4′-fluorophenyl)-imidazo[2,1-*b*] [1,3,4] thiadiazole derivatives as potent anticancer agents. Eur. J. Med. Chem..

[13] Kumar S., Hegde M., Gopalakrishnan V., Renuka V.K., Ramareddy S.A., De Clercq E., Schols D., Gudibabande Narasimhamurthy A.K., Raghavan S.C., Karki S.S. (2014). 2-(4-Chlorobenzyl)-6-arylimidazo[2,1-*b*] [1,3,4] thiadiazoles: Synthesis, cytotoxic activity and mechanism of action. Eur. J. Med. Chem..

[14] Arjomandi O.K., Hussein W.M., Vella P., Yusof Y., Sidjabat H.E., Schenk G., McGeary R.P. (2016). Design, synthesis, and in vitro and biological evaluation of potent amino acid-derived thiol inhibitors of the metallo-β-lactamase IMP-1. Eur. J. Med. Chem..

[15] Romagnoli R., Baraldi P.G., Prencipe F., Balzarini J., Liekens S., Estévez F. (2015). Design, synthesis and antiproliferative activity of novel heterobivalent hybrids based on imidazo[2,1-*b*] [1,3,4] thiadiazole and imidazo[2,1-*b*] [1,3] thiazole scaffolds. Eur. J. Med. Chem..

[16] Kumar S., Gopalakrishnan V., Hegde M., Rana V., Dhepe S.S., Ramareddy S.A., Leoni A., Locatelli A., Morigi R., Rambaldi M. (2014). Synthesis and antiproliferative activity of imidazo[2,1-*b*] [1,3,4] thiadiazole derivatives. Bioorg. Med. Chem. Lett..

[17] Patel H.M., Sing B., Bhardwaj V., Palkar M., Shaikh M.S., Rane R., Alwan W.S., Gadad A.K., Noolvi M.N., Karpoormath R. (2015). Design, synthesis and evaluation of small molecule imidazo[2,1-*b*] [1,3,4] thiadiazoles as inhibitors of transforming growth factor-β type-I receptor kinase (ALK5). Eur. J. Med. Chem..

[18] Parrino B., Carbone A., Ciancimino C., Spanò V., Montalbano A., Barraja P., Cirrincione G., Diana P., Sissi C., Palumbo M. (2015). Water-soluble isoindolo[2,1-a] quinoxalin-6-imines: In vitro antiproliferative activity and molecular mechanism(s) of action. Eur. J. Med. Chem..

[19] Parrino B., Ullo S., Attanzio A., Cascioferro S., Spanò V., Carbone A., Montalbano A., Barraja P., Cirrincione G., Tesoriere L. (2018). Synthesis of 5H-pyrido[3,2-b] pyrrolizin-5-one tripentone analogs with antitumor activity. Eur. J. Med. Chem..

[20] Diana P., Stagno A., Barraja P., Montalbano A., Carbone A., Parrino B., Cirrincione G. (2011). Synthesis of the new ring system pyrrolizino[2,3-b] indol-4(5H)-one. Tetrahedron.

[21] Parrino B., Carbone A., Spanò V., Montalbano A., Giallombardo D., Barraja P., Attanzio A., Tesoriere L., Sissi C., Palumbo M. (2015). Aza-isoindolo and isoindolo-azaquinoxaline derivatives with antiproliferative activity. Eur. J. Med. Chem..

[22] Diana P., Stagno A., Barraja P., Carbone A., Parrino B., Dall’Acqua F., Vedaldi D., Salvador A., Brun P., Castagliuolo I. (2011). Synthesis of triazenoazaindoles: A new class of triazenes with antitumor activity. ChemMedChem.

[23] Parrino B., Ciancimino C., Carbone A., Spano’ V., Montalbano A., Barraja P., Cirrincione G., Diana P. (2015). Synthesis of isoindolo [1,4] benzoxazinone and isoindolo [1,5] benzoxazepine: Two new ring systems of pharmaceutical interest. Tetrahedron.

[24] Cascioferro S., Attanzio A., Di Sarno V., Musella S., Tesoriere L., Cirrincione G., Diana P., Parrino B. (2019). New 1,2,4-Oxadiazole Nortopsentin Derivatives with Cytotoxic Activity. Mar. Drugs.

[25] Parrino B., Attanzio A., Spanò V., Cascioferro S., Montalbano A., Barraja P., Tesoriere L., Diana P., Cirrincione G., Carbone A. (2017). Synthesis, antitumor activity and CDK1 inhibiton of new thiazole nortopsentin analogues. Eur. J. Med. Chem..

[26] Spanò V., Attanzio A., Cascioferro S., Carbone A., Montalbano A., Barraja P., Tesoriere L., Cirrincione G., Diana P., Parrino B. (2016). Synthesis and Antitumor Activity of New Thiazole Nortopsentin Analogs. Mar. Drugs.

[27] Carbone A., Parrino B., Di Vita G., Attanzio A., Spanò V., Montalbano A., Barraja P., Tesoriere L., Livrea M.A., Diana P. (2015). Synthesis and antiproliferative activity of thiazolyl-bis-pyrrolo [2,3-b] pyridines and indolyl-thiazolyl-pyrrolo [2,3-c] pyridines, nortopsentin analogues. Mar. Drugs.

[28] Parrino B., Carbone A., Di Vita G., Ciancimino C., Attanzio A., Spanò V., Montalbano A., Barraja P., Tesoriere L., Livrea M.A. (2015). 3-[4-(1H-indol-3-yl)-1,3-thiazol-2-yl]-1H-pyrrolo[2,3-b] pyridines, nortopsentin analogues with antiproliferative activity. Mar. Drugs.

[29] Meijer L.L., Garajova I., Caparello C., Le Large T.Y., Frampton A., Vasile E., Fune N., Kazemier G., Giovannetti E. (2019). Plasma miR-181a-5p Down-Regulation Predicts Response and Improved Survival After FOLFIRINOX in Pancreatic Ductal Adenocarcinoma. Ann. Surg..

[30] El Hassouni B., Li Petri G., Liu D., Cascioferro S., Parrino B., Hassan W., Diana P., Ali A., Frampton A.E., Giovannetti E. (2019). Pharmacogenetics of treatments for pancreatic cancer. Expert Opin. Drug Metab. Toxicol..

[31] Le Large T.Y.S., El Hassouni B., Funel N., Kok B., Piersma S.R., Pham T.V., Olive K.P., Kazemier G., van Laarhoven H.W.M., Jimenez C.R. (2019). Proteomic analysis of gemcitabine-resistant pancreatic cancer cells reveals that microtubule-associated protein 2 upregulation associates with taxane treatment. Ther. Adv. Med. Oncol..

[32] Giovannetti E., van der Borden C.L., Frampton A.E., Ali A., Firuzi O., Peters G.J. (2017). Never let it go: Stopping key mechanisms underlying metastasis to fight pancreatic cancer. Semin. Cancer Biol..

[33] Le Large T.Y.S., Bijlsma M.F., Kazemier G., van Laarhoven H.W.M., Giovannetti E., Jimenez C.R. (2017). Key biological processes driving metastatic spread of pancreatic cancer as identified by multi-omics studies. Semin. Cancer Biol..

[34] Iwamura T., Caffrey T.C., Kitamura N., Yamanari H., Setoguchi T., Hollingsworth M.A. (1997). P-selectin expression in a metastatic pancreatic tumor cell line (SUIT-2). Cancer Res..

[35] Deer E.L., Gonzalez-Hernandez J., Coursen J.D., Shea J.E., Ngatia J., Scaife C.L., Firpo M.A., Mulvihill S.J. (2010). Phenotype and Genotype of Pancreatic Cancer Cell Lines. Pancreas.

[36] Maftouh M., Avan A., Funel N., Frampton A.E., Fiuji H., Pelliccioni S., Castellano L., Galla V., Peters G.J., Giovannetti E. (2014). miR-211 modulates gemcitabine activity through downregulation of ribonucleotide reductase and inhibits the invasive behavior of pancreatic cancer cells. Nucleosides Nucleotides Nucleic Acids.

[37] Avan A., Crea F., Paolicchi E., Funel N., Galvani E., Marquez V.E., Honeywell R.J., Danesi R., Peters G.J., Giovannetti E. (2012). Molecular Mechanisms Involved in the Synergistic Interaction of the EZH2 Inhibitor 3-Deazaneplanocin A with Gemcitabine in Pancreatic Cancer Cells. Mol. Cancer Ther..

[38] Avan A., Caretti V., Funel N., Galvani E., Maftouh M., Honeywell R.J., Lagerweij T., Van Tellingen O., Campani D., Fuchs D. (2013). Crizotinib inhibits metabolic inactivation of gemcitabine in c-Met-driven pancreatic carcinoma. Cancer Res..

[39] Ebos J.M.L., Lee C.R., Cruz-Munoz W., Bjarnason G.A., Christensen J.G., Kerbel R.S. (2009). Accelerated metastasis after short-term treatment with a potent inhibitor of tumor angiogenesis. Cancer Cell.

[40] Carbone A., Parrino B., Cusimano M.G., Spanò V., Montalbano A., Barraja P., Schillaci D., Cirrincione G., Diana P., Cascioferro S. (2018). New Thiazole Nortopsentin Analogues Inhibit Bacterial Biofilm Formation. Mar. Drugs.

[41] Boosa V., Bilakanti V., Velisoju V.K., Gutta N., Inkollu S., Akula V. (2018). An insight on the influence of surface Lewis acid sites for regioselective CH bond C3-cyanation of indole using NH4I and DMF as combined cyanide source over Cu/SBA-15 catalyst. Mol. Catal..

[42] Sciarrillo R., Wojtuszkiewicz A., Kooi I.E., Gómez V.E., Boggi U., Jansen G., Kaspers G.-J., Cloos J., Giovannetti E. (2016). Using RNA-sequencing to Detect Novel Splice Variants Related to Drug Resistance in In Vitro Cancer Models. J. Vis. Exp..

[43] Massihnia D., Avan A., Funel N., Maftouh M., van Krieken A., Granchi C., Raktoe R., Boggi U., Aicher B., Minutolo F. (2017). Phospho-Akt overexpression is prognostic and can be used to tailor the synergistic interaction of Akt inhibitors with gemcitabine in pancreatic cancer. J. Hematol. Oncol..

[44] Bray F., Ferlay J., Soerjomataram I., Siegel R.L., Torre L.A., Jemal A. (2018). Global cancer statistics 2018: GLOBOCAN estimates of incidence and mortality worldwide for 36 cancers in 185 countries. CA Cancer J Clin..

[45] Caparello C., Meijer L.L., Garajova I., Falcone A., Le Large T.Y., Funel N., Kazemier G., Peters G.J., Vasile E., Giovannetti E. (2016). FOLFIRINOX and translational studies: Towards personalized therapy in pancreatic cancer. World J. Gastroenterol.

[46] Kleeff J., Korc M., Apte M., La Vecchia C., Johnson C.D., Biankin A.V., Neale R.E., Tempero M., Tuveson D.A., Hruban R.H. (2016). Pancreatic cancer. Nat. Rev. Dis. Primers.

